# An archived taxonomic website: *Millipedes of Australia*

**DOI:** 10.3897/BDJ.7.e36385

**Published:** 2019-06-25

**Authors:** Robert Evan Mesibov

**Affiliations:** 1 Unaffiliated, West Ulverstone, Tasmania, Australia Unaffiliated West Ulverstone, Tasmania Australia

**Keywords:** Australia, Diplopoda, locality data, taxonomy, website

## Abstract

The taxonomic parts of the privately maintained *Millipedes of Australia* website (2006-2019), now offline, have been archived in Zenodo and are no longer being updated. Core taxonomic information about the Australian millipede fauna is now available on *MilliBase*, a global taxonomic resource for millipedes. Most of the locality records for named, native Australian millipedes formerly available as downloads on the *Millipedes of Australia* website are now accessible through the Atlas of Living Australia and the Global Biodiversity Information Facility.

## Introduction

The *Millipedes of Australia* website (*MoA*) was built as an alternative to the online Diplopoda checklist in the Australian Faunal Directory (AFD; https://biodiversity.org.au/afd/home). I compiled the first version of the AFD millipede list in 2002, but as a millipede taxonomist in the early 2000s I wanted a single source for more detailed taxonomic information on the Australian fauna and for detailed species locality information. *MoA* went online in 2006 as a privately maintained resource hosted by the Queen Victoria Museum and Art Gallery in Launceston, Tasmania, Australia. It contained synonymies for genera and species, as well as information about primary and secondary type specimens.

Over the next few years I upgraded the format and content of *MoA* and in 2010 moved it to a private domain, "polydesmida.info". Localities were first available in 2007 as markers on an interactive map of Australia. In 2012 the interactive map was replaced by downloadable CSV and KML files, grouped by genus. Locality records for named species were derived from specimen records in museum databases. For a discussion of the museum sources and how the records were checked and processed, see Mesibov (2013).

Two *MoA* features were available for short periods only. One was an interactive gallery of gonopod images for named, native Australian Polydesmida. The second was a set of webpages explaining the shell scripts I had written to generate both the static webpages in *MoA* and the downloadable CSV and KML files. All taxonomic and locality data were stored in tables of structured text rather than in a database. Whenever I updated the tables, the scripts were used to build new static webpages or new CSV and KML files to replace out-of-date versions.

In 2015 I added an illustrated key to the millipede orders known in Australia (native and introduced), and in 2016 I added links on the *MoA* bibliography page to non-paywalled, online versions of cited literature.

In March 2019 *MoA* was upgraded from XHTML 1.0 Strict to HTML5. The locality information and files were removed. A note on the top-level pages explained that locality records for named, native Tasmanian millipedes had been provided to the Atlas of Living Australia (ALA) (https://collections.ala.org.au/public/show/dr444), and that most locality records for named, native non-Tasmanian millipedes in *MoA* could now be found in ALA or the Global Biodiversity Information Facility (https://www.gbif.org).

On 17 May 2019 I removed *MoA* from "polydesmida.info" and archived the website in Zenodo as a freely available resource, no longer updated. Most of the taxonomic information in MoA now appears in *MilliBase* (http://www.millibase.org), a global taxonomic database for millipedes built on the Aphia platform (Vandepitte et al. 2015) and hosted by the Flanders Marine Institute. More information from *MoA* is expected to be added to *MilliBase* in coming years.

## Description of the archived website

*MoA* in Zenodo is a ZIP archive containing a CSS file (external style sheet), an image folder with 16 small images, eight top-level HTML pages, a "genus" folder with 22 HTML webpages providing genus-level taxonomic details and a "species" folder with 114 HTML pages providing species-level taxonomic details. The eight top-level pages are:

**index.html.** Introduction to the archived website, with a chart showing the cumulative number of described, native Australian millipede species, 1844-2019.

**catalogue.html.** Systematically arranged checklist of genera and species, with each name linked to the appropriate location on a page in the "genus" or "species" folders. Names include author and year. Species names are followed by distribution as State or Territory abbreviations (in square brackets). Includes species *incertae sedis*. The higher classification follows Shelley (2003).

**genus.html.** Alphabetical list of 152 genera (111 accepted), with each name linked to the appropriate location on a page in the "genus" folder. Names include author and year, and are followed by the number of valid species and millipede order, and synonyms (if any) with author and year.

**species.html.** Alphabetical list of 566 species (539 accepted), with each name linked to the appropriate location on a page in the "species" folder. Names include author and year, and are followed by distribution as State or Territory abbreviations, and synonyms (if any) with author and year.

**bibliography.html.** Alphabetical and chronological list of 292 taxonomic references cited on pages in the "genus" and "species" folders, and of articles mentioned on the introduced species page (introduced.html). Each reference is followed by a link to a freely available online version (if one exists), and by the date of the most recent link check.

**museums.html.** Alphabetical list of acronyms for type specimen repositories cited on pages in the "genus" and "species" folders. Each acronym is followed by a repository name and location.

**introduced.html.** Systematically arranged checklist of 24 millipede species known to have been introduced to Australia and its territories. For each State or Territory with records, the source of the record is given (publication or museum record).

**key2orders.html.** An illustrated, one-page key to the eight native and one introduced millipede orders found in Australia.

**Figure 1.**
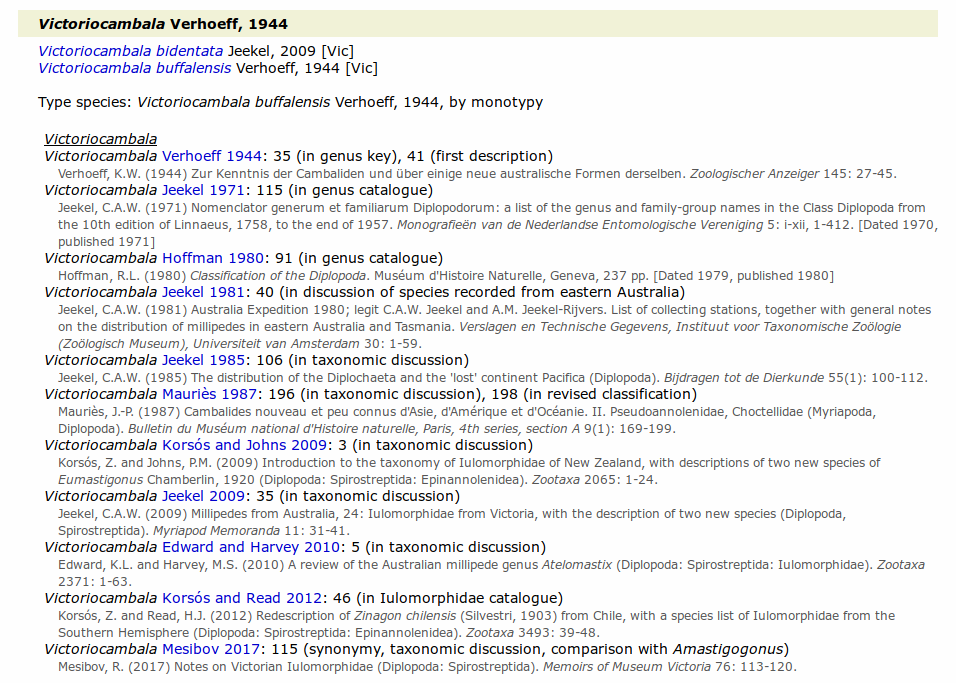
Screenshot of information on the genus *Victoriocambala* Verhoeff, 1944 from the Iulomorphidae webpage (iulomorphidae.html).

Each page in the "genus" folder lists genera within a higher taxon (family, subfamily or tribe) together with generic synonyms. Each accepted genus is accompanied by a list of its accepted species, with each species name linked to the appropriate location on a page in the "species" folder and followed by author and year, and distribution as State or Territory abbreviations (in square brackets). Each genus name (including synonyms) is followed by a chronological synonymy, with reference links to the bibliography page, relevant page references and their significance, and the full reference citation. For each genus name (and synonym) the type species and the method of type designation are given (Fig. 1).

**Figure 2.**
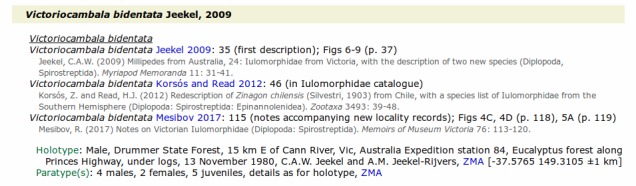
Screenshot of information on the species *Victoriocambala bidentata* Jeekel, 2009 from the *Victoriocambala* webpage (victoriocambala.html)

Each page in the "species" folder lists species (or subspecies) within a single genus, together with species synonymies presented as for genus names, and followed by a listing of known primary and secondary types. The type list includes the following items (if known): number and gender of specimens, published or other collection details, repository abbreviation linked to the MoA museums page and repository catalog number. Latitude/longitude estimates are provided for type localities published without georeferences, and some uncertain type listings are followed by explanatory notes (Fig. 2).

## Data resource

https://doi.org/10.5281/zenodo.2885742
